# Perceptions of inhibitors and facilitators for adhering to hypertension treatment among insured patients in rural Nigeria: a qualitative study

**DOI:** 10.1186/s12913-014-0624-z

**Published:** 2014-12-10

**Authors:** Aina O Odusola, Marleen Hendriks, Constance Schultsz, Oladimeji A Bolarinwa, Tanimola Akande, Akin Osibogun, Charles Agyemang, Gbenga Ogedegbe, Kayode Agbede, Peju Adenusi, Joep Lange, Henk van Weert, Karien Stronks, Joke A Haafkens

**Affiliations:** Department of Global Health, Academic Medical Center, University of Amsterdam, Amsterdam Institute for Global Health and Development, Amsterdam, The Netherlands; Department of Epidemiology and Community Health, University of Ilorin Teaching Hospital, P.M.B. 1459, postal code 240001 Ilorin, Nigeria; Department of Community Health, Lagos University Teaching Hospital, P.M.B.12003, Surulere, Lagos Nigeria; Department of Public Health, Academic Medical Center, University of Amsterdam, Meibergdreef 9, Amsterdam, 1105 AZ The Netherlands; Division of Health and Behavior, Center for Healthful Behavior Change, Department of Population Health, NYU School of Medicine, New York, NY 10016 USA; Ogo Oluwa Hospital, 64/65 Ahmadu Bello Way, Bacita, Kwara State Nigeria; Hygeia Community Health Care, Hygeia HMO, 13B Idejo Street, Lagos, Nigeria; Department of General Practice, Academic Medical Center, University of Amsterdam, Meibergdreef 9, Amsterdam, 1105 AZ The Netherlands

**Keywords:** Insured hypertension care, Perceptions, Inhibitors, Facilitators, Adherence, Health awareness

## Abstract

**Background:**

Universal health care coverage has been identified as a promising strategy for improving hypertension treatment and control rates in sub Saharan Africa (SSA). Yet, even when quality care is accessible, poor adherence can compromise treatment outcomes. To provide information for adherence support interventions, this study explored what low income patients who received hypertension care in the context of a community based health insurance program in Nigeria perceive as inhibitors and facilitators for adhering to pharmacotherapy and healthy behaviors.

**Methods:**

We conducted a qualitative interview study with 40 insured hypertensive patients who had received hypertension care for > 1 year in a rural primary care hospital in Kwara state, Nigeria. Supported by MAXQDA software, interview transcripts were inductively coded. Codes were then grouped into concepts and thematic categories, leading to matrices for inhibitors and facilitators of treatment adherence.

**Results:**

Important patient-identified facilitators of medication adherence included: affordability of care (through health insurance); trust in orthodox “western” medicines; trust in Doctor; dreaded dangers of hypertension; and use of prayer to support efficacy of pills. Inhibitors of medication adherence included: inconvenient clinic operating hours; long waiting times; under-dispensing of prescriptions; side-effects of pills; faith motivated changes of medication regimen; herbal supplementation/substitution of pills; and ignorance that regular use is needed. Local practices and norms were identified as important inhibitors to the uptake of healthier behaviors (e.g. use of salt for food preservation; negative cultural images associated with decreased body size and physical activity). Important factors facilitating such behaviors were the awareness that salt substitutes and products for composing healthier meals were cheaply available at local markets and that exercise could be integrated in people’s daily activities (e.g. farming, yam pounding, and household chores).

**Conclusions:**

With a better understanding of patient perceived inhibitors and facilitators of adherence to hypertension treatment, this study provides information for patient education and health system level interventions that can be designed to improve compliance.

**Trial registration:**

ISRCTN47894401.

**Electronic supplementary material:**

The online version of this article (doi:10.1186/s12913-014-0624-z) contains supplementary material, which is available to authorized users.

## Background

Cardiovascular disease (CVD) is a leading cause of death globally [1,2]. Hypertension is a major risk factor for CVD [3]. Once rare in SSA, hypertension and its related complications are increasingly common in the region [4]. Treatment with medication and behavioral changes (reduction in dietary salt intake, weight reduction, moderation of alcohol intake and increased physical activity) can greatly reduce blood pressure (BP) and the risk of CVD-related mortality among people with hypertension [5]. In SSA, CVD prevention is a recent development [6], and hypertension detection, treatment and control rates are generally low [7]. The absence of affordable community-based primary care services has been identified as a major obstacle to effective hypertension treatment in the region [8]. But several countries in SSA are now developing programs that provide such services [9,10]. Hypertension is a chronic condition that requires lifelong adherence to pharmacotherapy and healthy behaviors. For many patients, long-time adherence to hypertension treatment is a problem [11-16]. There is evidence that hypertension care is more effective if it includes educational interventions that address barriers to adherence [12,17,18]. Theoretical frameworks underlying various models for patient education propose that, in order to be effective, education should be patient-centered, and tailored to patients’ (cultural) views about the condition and the treatment [17-19]. In high income countries, where access to affordable care is generally available, many (mostly qualitative) studies have investigated patients’ perspectives on adherence to hypertension treatment in different populations [13,20-22]. A recent systematic review of 53 of those studies indicated that common patient-related factors for non-adherence to antihypertensive medication across countries and ethnic groups include patients’ beliefs that medication is unnecessary when symptoms of hypertension or stress disappear, a dislike of medications, fear of addiction, and the experience of side effects [22]. Data from such studies have provided content for educational interventions to improve adherence and health outcomes in patients with hypertension in the USA and Europe [23-25]. Non-adherence to hypertension treatment can have serious consequences for the patients, in terms of lost opportunities for health improvements, and for the health care systems, in terms of wasted financial and human resources. New programs that provide universal primary care coverage are emerging in SSA [26]. So far, few studies in the region have investigated perceptions of hypertension treatment among patients who are covered by health insurance [27]. Hence, it is important to fill this gap [28]. Nigeria is one of the countries in SSA where initiatives are being taken to introduce private or public community based health insurance plans [29]. The estimated prevalence rates of hypertension in Nigeria are 19.3% for rural areas, 36.6% for semi-urban areas, 42% for urban areas, and from 12.4%-to-34.8% for the country as a whole [29-32]. Studies of patients’ perceptions of hypertension treatment have been carried out in several settings and communities in Nigeria [15,16,33-36], but not among patients who were covered by health insurance. Understandably, many of these studies concluded that patients perceived financial constraints as the major reason for not adhering to treatment [16,37,38]. The present study was conducted in the context a subsidized community based health insurance program that covers the costs of primary and limited secondary health care and medications, for low income residents of rural communities in Kwara state, the 9th poorest among Nigeria’s 36 states. A recent population-based study that was conducted in Kwara reported hypertension awareness, treatment and control rates of 8%, 5% and 3%, respectively [29]. As a first stage of a larger project [39], we conducted a qualitative study among hypertensive patients who participated in the community based health insurance program in Kwara state to explore their views on hypertension management. The specific research questions of the study were as follows: 1) what are patients’ views on factors that may inhibit or facilitate adherence to prescribed medications?; and 2) what are patients’ views on factors that may inhibit or facilitate adherence to behavioral recommendations?

## Methods

We used a qualitative research design and individual interviews with open-ended questions to explore patients’ perceptions on inhibitors and facilitators for adhering to hypertension treatment [40].

### Setting and participants

For this study we recruited patients who had enrolled in the Hygeia Community Health Care Plan and were treated for hypertension at Ogo Oluwa Hospital, which is located in Bacita, a small rural town in Kwara state, Nigeria.

The Hygeia Community Health Care Plan, now called the Kwara State Community Health Program, is financed by an international development organization – the Health Insurance Fund (HIF) [41,42]. This health care plan was launched in 2007 to provide subsidized community based health insurance for low- and middle income groups in western Nigeria (Lagos) and north-central Nigeria (Kwara state). The insurance package covers primary and limited secondary care, including hypertension treatment.

Ogo Oluwa Hospital is one of the first clinics in Kwara state that joined the Hygeia Community Health Care Plan. It is a large, busy private hospital that has offered primary and secondary care to people from the Bacita area for over 25 years. In the 1990s, Bacita was a busy commercial center that hosted the then thriving but later moribund state-owned Nigeria Sugar Company. The original population of Bacita is Nupe, but the Sugar Company attracted other Nigerians of various ethnic origins, particularly the Yoruba. Although the Sugar Company went out of business, a substantial majority of the economic migrants of the time settled permanently in the Bacita area. Therefore, Yoruba is now widely spoken alongside the native Nupe. Currently considered a poor community, the predominant livelihood activities of the inhabitants are farming, petty trading, artisanship, hunting and fishing.

Ogo Oluwa Hospital is owned and managed by an experienced General Practitioner, assisted at that time by about 2 other doctors, 10 nurses, 3 pharmacy staff, 4 laboratory staff and 4 records staff. At the start of this study, about 400 of the hospital’s patients were treated for hypertension and attended clinics on a monthly or bi-monthly basis. Most of them had joined the Hygeia Community Health Care Plan. About two-thirds resided within the town at an average distance of 10 to 20 minutes travel time to the hospital; the remaining third were living in some distantly located surrounding villages.

The inclusion criteria for this study were as follows: having Hygeia Community Health Care Plan; diagnosed with hypertension (systolic BP ≥ 140 mmHg and/or diastolic BP ≥ 90 mmHg); in treatment for hypertension at Ogo Oluwa Hospital for ≥ 12 months; having been prescribed antihypertensive medication for ≥ 12 months; aged 18 years or over; and being prepared to give verbal (recorded) informed consent. Since pregnancy induced hypertension has a different course and etiology, pregnant or lactating hypertensive females were excluded from participation in this study. We also sought equal participation of men and women.

In qualitative interview studies, data or thematic saturation is a criterion for determining sample size [43,44]. This means that the number of respondents is sufficient if interviews with new respondents do not yield new themes. Fifteen interviews is generally enough to achieve saturation [43,44]. On the basis of these considerations and the inclusion criteria, we selected a purposeful sample of 40 patients from the case file records of Ogo Oluwa Hospital and invited them to the study. None of the invited patients declined. Patients who agreed to participate were reminded about the appointment some days prior to date of interview. At the beginning of each interview session, patients were asked to give audio-recorded informed consent and were assured of confidentiality. Travel expenses incurred for study visits outside usual clinic days were reimbursed.

### Interview guide and data collection

To explore patients’ perceptions of hypertension treatment we developed a semi-structured interview guide. The guide built on instruments that were used in previous qualitative interview studies of patient perceptions of hypertension [13,45] and it was adapted to the specific context and purpose of this study. The interview guide was edited in English and translated into Yoruba and Nupe by native speakers. Eight main topic areas of the interview guide that referred to hypertension treatment were: knowledge and personal views about hypertension; access to care; the role of health insurance; sources of information about hypertension; views on (prescribed) medications; views of behavioral adaptations; satisfaction with care; and general views on CVD prevention (Additional file 1). Information on the socio-demographic characteristics of the participants was collected through close-ended questions. Information on the participants’ recent health related outcomes, (blood pressure, co-morbidities), was collected from the respondents’ case file records. Data collection and analysis are continuous and simultaneous iterative processes in qualitative studies [46]. In this study, we held a first set of 30 interviews between July and October 2010 and a second set of 10 interviews between November and December 2010. The specific aim of the last set of interviews was to explore a number of emerging themes that were identified through the analysis of the first 30 interviews.

The researcher (AOO) conducted the interviews at Ogo Oluwa Hospital. He is not from the study area and he did not know the participants, prior to the interview. He made his profession, researcher with a medical background (MD), known to the participants.

Each of the first set of interviews lasted 120 minutes on average, and the second set lasted about 60 minutes each. All interviews were recorded, transcribed and translated into English by the researcher and an assistant.

### Data analysis

The interview transcripts were analyzed, using steps and coding procedures of the method of qualitative data analysis that was developed by Strauss and colleagues (grounded theory) [47]. Briefly, this inductive method begins with the assignment of a series of open codes to the transcripts of individual interviews, which are then grouped into clusters (concepts) in order to make them more workable. From these concepts, broader categories and subcategories are generated, through a process of constant comparison or verification. The ultimate outcome is a data-based matrix of concepts and (sub-) categories, which serve as an input for developing theoretical or conceptual frameworks.

We imported all interview transcripts into MAXQDA and we used this qualitative data analysis software to support consistent data analysis, processing, ordering and comparison of the results at different stages [48]. Underneath we provide some more detail about the process of data analysis that was adopted in the present study.

First, in each of the interview transcripts, sections containing information on respondents’ ideas on hypertension and hypertension treatment (medications and behavioral changes) were identified, and inductively coded. Similar codes were assigned to text fragments that reflected the same idea. These coding activities yielded a codebook that included all the codes that were created during the analysis of the individual interview transcripts. To conclude this stage we reviewed the code book to eliminate duplicates and to inspect if the remaining codes captured similar concepts so that they could be grouped together. The goal was to establish a smaller set of mutually exclusive conceptual codes that expressed the meaning of the underlying codes without loss of the original content. The identified “concepts”, and underlying text fragments were then scrutinized and grouped under four major themes referring to our research questions; inhibitors and facilitators of adherence to medication and behavioral recommendations. Subsequently, thematic coding procedures were used to group the concepts under each of the four major themes into categories and sub-categories. Results of these multi-level coding procedures were summarized into matrices. (See Additional file 2 for an example).

### Trustworthiness

The researcher (AOO) selected the sections of the interview transcripts that served as an input for the data analysis. To increase reliability and trustworthiness of the analysis, the following steps were taken:

1. To establish a consistent open coding procedure, two members of the research team (AOO and JAH) coded fragments of 15 interviews independently. Results were compared and if differences occurred (for instance with respect to the most plausible naming of a code) they were resolved through discussion and by going back to the original data.
2. To establish the (short) list of conceptual codes, two members of the team (AOO and JAH) independently reviewed the code book and created relevant concepts. Differences of opinion with respect to the clustering of codes or the naming of concepts were resolved by discussion and by going back to the original data.
3. The categorization of concepts under the main themes (inhibitors and facilitators of adherence) was done by AOO and supervised by JAH, who made sure that every decision was plausible and could be justified.
4. The preliminary results of the data analysis were based on 30 interviews. Overlooking these results, AOO and JAH acknowledged that some additional interviews (10) were needed to further confirm or explore some of the themes and concepts that had emerged from this preliminary analysis, so that thematic saturation could be reached. Examples of such themes were related to the perceived role of religion on (non-) adherence to medication and patients’ ideas about “local practices” that can inhibit or enhance healthy eating habits and physical exercise.
5. Based on the analysis of the last set of interviews some new conceptual codes were added to the original list, where appropriate. The researcher returned to the transcripts of the first set of interviews to check if any of these new codes could also be applied here.
6. Thematic coding was done by AOO and JAH. They reviewed the conceptual coding trees for each of the four major themes (research questions) and independently categorized those concepts into categories and subcategories. Differences in opinion were resolved by discussions and by checking the underlying data. At several occasions other members of the research team (KS, CS) asked critical questions as regards the categorization of the concepts, and this led to some adaptations in the final ordering and presentation of the categories and sub categories and consequently the results.
7. The final results of the analyses that are presented in the Tables in this paper were reviewed by all authors.

### Ethics

This study is part of a larger project that aims to develop and evaluate cardiovascular health education program for insured patients with hypertension in rural Nigeria [39]. Ethical approval was obtained on 30th March, 2010 from University of Ilorin Teaching Hospital prior to the start of the study in July. Guidelines for quality assurance of qualitative research in health from the Academic Medical Center were used to ensure appropriate conduct of the study [49]. COREQ criteria for reporting qualitative interviews were used as a guideline for reporting this study [50].

## Results

We interviewed 40 patients. Half of them had controlled BP – systolic BP < 140 mmHg and diastolic BP < 90 mmHg, and the other half uncontrolled BP – systolic BP ≥ 140 mmHg and/or diastolic BP ≥ 90 mmHg (Table 1). Most were older than 50 years. All were rural dwellers, 80% earned less than 5 USD per day, and 70% had none or only primary school education, almost all (92%) lived in poor housing conditions and most (75%) were farmers, artisans or traders. All were religious, 62.5% practiced Christianity and 37.5% practiced Islam.

**Table 1:** **Socio-demographic and clinical characteristics of respondents (n = 40)**

**Characteristics**	**N (%)**
**Age group (years)**	
30-50	9 (22.5%)
50-70	26 (65%)
70-90	5 (12.5%)
**Gender**	
Male	16 (40%)
Female	24 (60%)
**Educational level**	
None or primary education	28 (70%)
Secondary education	9 (22.5%)
Tertiary education	3 (7.5%)
**Ethnicity**	
Nupe	6 (15%)
Yoruba	31 (77.5%)
Others	3 (7.5%)
**Religion**	
Christianity	25 (62.5%)
Islam	15 (37.5%)
**Marital status**	
Married	38 (95%)
Widowed	2 (5%)
**Employment status**	
Unemployed	1 (2.5%)
Self-employed*	39 (97.5%)
**Income level (USD per day)**	
Less than 2	23 (57.5%)
2 – 4	9 (22.5%)
More than 5	8 (20%)
**Health insurance status**	
Insured (Hygeia Community Health Care Plan)	40 (100%)
**Hypertension control status**	
Controlled	20 (50%)
Uncontrolled	20 (50%)
**Duration of hypertension history (years)**	
1 – 5	28 (70%)
5 – 10	7 (17.5%)
Above 10	5 (12.5%)
**Co-morbid conditions**	
Diabetes	7 (17.5%)
Others (osteoarthritis, peptic ulcer disease)	7 (17.5%)
None	26 (65%)

*Most frequent employment types: artisanship/farming/trading/hunting/fishing.

### Factors inhibiting and facilitating medication adherence

Some patients said they did not always take their medications according to prescription. Frequently mentioned patterns of “self-regulation” included reducing the daily dosage, skipping medications under specific circumstances and discontinuing medications for several days or weeks. None had stopped using medications all together. Patients’ accounts of factors that inhibited them from using their pills as prescribed could be classified into five main categories (Table 2):

**Table 2:** **Factors inhibiting adherence to medications: thematic matrix of categories, subcategories and concepts (n = 40)**

**Category**	**Sub-category**	**Concepts**
1) Healthcare related factors	Inflexible clinic hours	Clinic visits always coincide with religious worship time [n = 3]*
		Works/farm during clinic hours [n = 10]
		Difficult to refill drugs for longer periods when travelling out of town [n = 6]
	Long waiting time	Less time available for business or other important activities [n = 31]
	Logistics/ travel costs constraints	Residence too far from clinic [n = 4]
	Medications out of stock	Under dispensed prescriptions [n = 11]
		Prescribed pills not dispensed [n = 3]
2) Patient related factors	Poor knowledge about hypertension	Hypertension is curable, transient [n = 23]
		Feels well, no symptoms, so don’t use pills [n = 3]
3) Medication related factors	Adverse effects, side effects	Abandon pills to avoid intolerable effects [n = 7]
	Complexity of prescription regimes	Too many pills prescribed, too frequent dosing to follow [n = 6]
	Substitution/supplementation of prescribed medicines	Takes herbal drugs alongside prescribed pills [n = 5]
		Takes herbal drugs in place of prescribed pills [n = 2]
4) Religion related factors	Medication use discouraged by faith practice	Believes in faith healing [n = 2]
		Dosing frequency incompatible with faith practice (fasting) [n =13]
5) Social factors	None or poor social support	Wife lacks husband’s approval (which is) mandatory for outings – clinic visits [n = 1]

*n refers to the number of respondents whose perceptions contribute to the corresponding concepts.

1. Healthcare related factors: The way in which hypertension care was organized was perceived as one inhibitory factor for medication adherence. In general patients at Ogo Oluwa hospital are required to attend hypertension clinic once a month for follow-up and medication refills. The hypertension clinic is always held at fixed hours on Fridays. Other obligations such as attending Muslim praying hours (Fridays), work, household chores or being on a trip out of town will sometimes prevent patients from meeting their appointments at these fixed hours. The distance to the clinic was also mentioned as a reason for missing appointments. Highlighting the resultant financial challenge, a 50 year old woman with uncontrolled hypertension [ID6] said – *visiting [regularly] is problematic for me in the sense that the clinic is far from my residence and travel cost is prohibitive; I spend 500 naira (3USD) to get here and another 500 naira to go back home*. Long waiting times at the clinic were also cited as a reason for missing appointments. Asked how regularly he attends clinic, a 72 year old man with uncontrolled hypertension [ID27] replied – *visiting is difficult for me because of the many hours spent in clinic, which could be used on my business; in fact I once stopped coming for about 2 months when I realized my hypertension was ‘under control’.* Sometimes, the pharmacy is out of stock for particular drugs and not all prescribed medications can be dispensed. A 50 year old woman with controlled hypertension [ID11] responded – *sometimes, the pharmacy doesn’t have all the drugs prescribed so they write it in paper for me to buy or come back to collect them oftentimes days after being without pills to use*.
2. Patient related factors: patients’ own views such as the idea that hypertension is a transient, curable condition also emerged as a consideration for deviating from the prescribed medication. Responding to a question on how long she expects to continue to use hypertension medication, a 64 year old woman with uncontrolled hypertension [ID13] replied – *surely, hypertension can be cured, one cannot continue to use drugs all days of one’s life; with prayers, in no time it will go.*
3. Medication related factors: side-effects experienced and the complexity of prescribed regimens also emerged as adherence inhibitors. A 55 year old woman with uncontrolled hypertension [ID38] responded *– I am okay with everything but the helplessness of the doctor about the serious side-effects that I experience with my drugs; I sometimes miss my clinic appointments because of these side-effects.* A 50 year old woman with uncontrolled hypertension [ID6] responded – *if the pills can be formulated such that fewer pills will do the work of the 8 pills that I currently take daily perfectly; then fewer pills are preferred as they are easier for me to manage.* Some patients mentioned incompatibility between religious fasting and regular pills use, and some others unilaterally substituted or supplemented prescribed pills with herbal remedies without recourse to their doctor. A participant [ID11] responded – *in order to observe my religious obligation (Ramadan fast), I usually skip my afternoon dose for the pills I normally take three times a day although I do not inform my doctor about this.* Asked what other treatments she uses, a 55 year old female participant with uncontrolled hypertension [ID16] responded – *apart from the pills given to me by my doctor, I also take ‘bitter leaf water’; it is said to be good for hypertension.*
4. Religious factors: the potential inhibitory influence of religion on adherence manifested in the accounts of some patients who strongly believe in faith healing. A woman with controlled hypertension [ID37] stated – *I use only the drugs prescribed to me, but ordinarily, as a member of Christ Apostolic Church Christian faith, I really do not use drugs if not that this is really important; I believe in faith healing.*
5. Social support: sometimes, lack of social support was an inhibitory factor for hypertension clinic visits. For instance, a husband (family head) may sometime disapprove his wife’s outings for reasons that may be personal. The wife of a religious leader (an Imam), with uncontrolled hypertension [ID6] responded – *I usually get a month’s supply of drugs from the pharmacy, but for reasons best known to him my husband would sometimes not allow me to come to the clinic and I cannot come without his permission.*

Table 3 shows respondents’ perceptions of factors that can facilitate medication adherence in five main categories:

**Table 3:** **Factors facilitating adherence to medications: thematic matrix of categories, subcategories and concepts (n = 40)**

**Category**	**Sub-category**	**Concepts**
1) Healthcare related factors	Affordability of quality ‘Hygeia Community Health Care’ Plan	Free access to ‘good’ Hygeia community health care “obliges” and encourages compliance with pills [n = 30]*
		Free Hygeia community health care is timely blessing from God and conscientious pill use maximizes this blessing [n = 5]
	Appreciation of healthcare provider	Impressive professional and social reputation of Doctor stimulates compliance [n = 12]
	Approachability of healthcare provider	The listening, concerned doctor [n = 29]
		Doctor’s willingness to dose pills relevant to circumstances (e.g. fasting period) [n = 10]
	Availability of medication	Pills free and always available [n = 30]
2) Patient related factors	Perceived dreaded nature of hypertension	Hypertension is dangerous and can kill [n = 38]
3) Medication related factors	Perceived efficacy of orthodox (western) medicines	White man’s pills work better than traditional medicines (herbs) [n = 20]
4) Religion related factors	Faith related support	Prayer makes pills work well [n = 19]
		Motivation from health counseling at faith meetings/services [n = 6]
5) Social support factors	Supportive and concerned family	Family members remind patient and monitor pill use [n = 10]
	Peer support	Motivation from other ‘positive living’ hypertensive patients [n = 4]

*n refers to the number of respondents whose perceptions contribute to the corresponding concepts.

1. Health care related factors; four sub-categories emerged from this category namely: affordability of care; appreciation of healthcare provider; thrust in orthodox medicines; and availability of prescribed pills. Some patients view the virtually free Hygeia Community Health Care Plan, previously unavailable in that community as a good and timely blessing from God. This encouraged them to adhere to their medications. A 61 year old man with controlled hypertension [ID22] stated – *this Hygeia insurance program is very useful for me and I do pray regularly for success for the operators of the program; the program manages my hypertension well and makes me healthier and stronger to work for my ‘daily bread’.*

The easy approachability of the doctor was also perceived as facilitating factor for medication adherence. A female participant with uncontrolled hypertension [ID3] responded – *last January when I was about to travel, I approached my Doctor and told him that I will be away for 2 months requesting enough drugs stock to last the period; this he willingly did, so I always take my drugs whether traveling or not.* Another, a 61 year old man with controlled hypertension [ID22] responded – *during the last fasting season, I approached my doctor 2 weeks earlier to seek advice on how to use my drugs, he decided to withdraw my ‘thrice a day’ pill and replace this with a ‘twice a day’ pill, and later reverted back to the ‘thrice a day’ drug after the month-long fasting.*

2) Patient related factors: responses grouped under this category show that knowledge and fear of complications of hypertension may motivate patients to comply with their medications. A 60 year old man with controlled hypertension [ID24] stated – *hypertension is something that brings death in different forms; one can be working and suddenly fall down and slump or become paralyzed. It is called ‘kosibale okan‘in Yoruba meaning ‘there’s no peace of mind’.* Another patient [ID29] similarly alluded to the danger of hypertension thus – *hypertension is dangerous and can kill suddenly; it shows no mercy*.
3) Medication related factors: many patients said they comply with their medications because they firmly believe orthodox ‘western’ medicines are effective, especially when compared with alternative ‘traditional’ medicines. A 70 year old man with uncontrolled hypertension [ID29] responded – *I am into herbs and I have used them severally in the past but they did not work like the white man’s medicine.*
4) Social support: some patients view reminders and monitoring by family members and support from peers with same condition as important factors that motivate compliance. Asked how carefully she uses her medicines, a 56 year old woman with controlled hypertension [ID37] replied – *I don’t miss my pills; if I do, my children will be annoyed with me, they monitor me closely to be sure I take my drugs regularly every day.*
5) Religious factors: many patients said prayer supported them in using and in improving the beneficial effect of their medications. Moreover, additional health education from religious leaders was also deemed important by some. A 62 year old man with controlled hypertension [ID25] said – *I have received enlightenment about hypertension from some radio programs and from my church too; they are useful advices and I usually followed them.*

### Factors inhibiting and facilitating adherence to behavioral recommendations

Managing hypertension requires a healthy behavior, including limited use of salt, weight reduction or maintenance, exercise, cessation of smoking and limited use of alcohol and other stimulants. Table 4 illustrates factors which inhibit achieving such healthy behaviors, in the eyes of our respondents.

**Table 4:** **Factors inhibiting compliance with behavioral measures: thematic matrix of categories, subcategories and concepts (n = 40)**

A) Salt consumption		
Category	Sub-category	Concepts
1) Local practices	Food preservation/conservation	Salt used to prevent decomposition of food [n = 3]*
		Salt preserved (canned or processed) foods used [n = 5]
	Food preparation	Salt, maggi used(in quantities) to cook/season meals [n = 14]
		Extra (table) salt added to already cooked meals [n = 2]
	Medicinal use of salt	Salted solution (salt water) used in treating stomach (abdominal) discomfort [n = 3]
2) Patient related factors	Poor hypertension knowledge	Ignorance of the influence of salt on hypertension [n = 2]
B) Weight control		
Category	Sub-category	Concepts
1) Local practices	Perceived relationship of weight to affluence/comfort/wealth	Societal views that ‘the fatter, the more affluent, the more comfortable’ [n = 12]
	Perceived relationship of weight to illness/disease	Societal view that ‘losing weight (slimming down) means serious illness’ [n = 13]
	Perceived relationship of weight to beauty	Societal view that ‘being fat enhances beauty/sexual attractiveness’ [n = 7]
	Perceived inheritableness of weight gain/obesity (family trait)	Heaviness (fatness) is inherited in my family; we usually have big sizes [n = 3]
	Perceived fattening tendency of local meals	Local meals heavily starch based and fattening [n = 5]
		Red palm oil and Groundnut oil are the only readily available cooking oils here [n = 11]
		Rampant local Goat breeding practice makes meat easily available and cheap [n = 2]
C) Exercise		
Category	Sub-category	Concepts
1) Local practices	Perceived needlessness of exercise	Societal view that ‘exercise is meant for the unengaged, unserious and greedy persons that rather walk to avoid travel costs’ [n = 5]
	Perceived danger of exercise for the aged	Societal view that ‘exercise is dangerous’ for older adults and the elderly [n = 7]
2) Patient related factors	Poor knowledge of relationship between exercise and hypertension	Perception that ‘exercise is dangerous’, makes hypertension worse [n = 5]
	Poor awareness on ‘how to exercise’	Ignorance about available and easy everyday exercise activities [n = 11]
D) Tobacco, alcohol, stimulants		
Category	Sub-category	Concepts
1) Local practices	Perceived benefits of tobacco (smoking)	Alcohol used in relieving stress/tension [n = 2]
	Perceived benefits of alcohol (local palm wine)	Palm wine makes vision clearer [n = 1]
	Perceived benefits of snuff (Nicotine powder)	Snuff useful in relieving stress and tension [n = 1]
	Perceived benefits of Kola nut consumption	Normal for older adults and the elderly to chew ‘Kola’ regularly; Kola nuts stimulate work [n = 2]

*n refers to the number of respondents whose perceptions contribute to the corresponding concepts.

1. Salt: while most participants said they had been informed by their doctor that they need to be prudent with the use of salt, our data also suggest that some patients were aware of the impact of salt on their BP. Local food practices may inhibit salt reduction; in some communities, salt is used to preserve food, particularly meat and fish against microbial decomposition over time. When asked what local customs encourage salt use, a 70 year old man with controlled hypertension [ID19] replied – *in some parts of the north where I come from, we use salt to preserve food and meat.* Food preparation practices can also inhibit compliance with advice to reduce salt. In the study region, adding salt and salt substitutes/seasoning agents like *maggi* (salt plus hydrogenated oil plus monosodium glutamate – MSG), *ajinomoto* (an MSG product) etc. has become standard practice while cooking. The consumption of canned (processed) foods, often salty is also increasingly common in the region. The previously fairly common cultural habit of drinking salted water in attempts to treat ‘undiagnosed’ abdominal discomfort of all sorts was practiced by some respondents. A 50 year old woman with uncontrolled hypertension [ID6] stated – *it is more or less customary for people to cook food with salt in this region,… and for some people who may sometimes have stomach upset, tradition demands that one should pour salt into a cup of water and drink to treat the ailment; I used to do this too but being better aware, have now stopped the practice*.
2. Weight control: participants mentioned a number of local or cultural practices that may prevent people from losing weight including the cultural perceptions; that large body sizes are associated with wealth, comfort or beauty; that weight loss is associated with disease; and that weight (*fatness*) is an unchangeable family trait. A 50 year old woman with uncontrolled hypertension [ID3] responded – *people often see being fat as synonymous with affluence, comfort and peace of mind; they also sometimes associate fatness in a woman with beauty. Some of us women prefer to be fat or robust believing that by so doing; we will be more attractive to our husbands and men generally. Some men too prefer to be plump so they will be liked by women.* A 63 year old man with controlled hypertension [ID30] said – *in this region*, *people often link weight loss to disease particularly if the slimming down is getting too much, suggesting that such might be due to disease; at other times they suggest that slim people are very miserly and would rather not spend money to eat well and get robust*. Highlighting the perceived link with inheritance, a 55 year old woman with uncontrolled hypertension [ID16] responded – *my friends and neighbors often say I was too fat, but I’m not bothered about such comments as I believe I inherited the trait from my mother; we are usually fat in my family.* Local food practices may also inhibit weight control. Some participants highlighted the difficulty in avoiding some of the main “fattening” ingredients of local popular dishes such as cassava, groundnut oil, (red) palm oil and meat. The first three ingredients are commonly grown by farmers in the community while meat is widely available due to popular goat breeding practice in the community whereby owning and breeding goats is a pastime that many families engage in.
3. Exercise: perceived inhibitors of exercising include ‘local practices’ and lack of information. Prevailing local or cultural views that exercise is “needless or useless’, dangerous, or incompatible with advancing age, may prevent people from being physically active. Exercise is sometimes regarded by people as an activity for unserious fellows, the unengaged or the miserly that rather walk habitually than pay for transportation costs to destinations. An elderly male patient with controlled hypertension [ID30] responded – *when people see me ‘walking to exercise’ they often slight me and make derogatory comments that one is greedy and would rather walk long distances than spend money on transportation.* Asked further how people see exercising or sporting activities, he replied – *some people see those who exercise as unserious people. Sports like playing football are only engaged by youths not elderly people like me.*

Several respondents said they were not aware or informed about positive effects of exercise on BP or easy ways to exercise. Insufficient knowledge makes some patients view exercise as dangerous for their hypertension. Some others are simply unfamiliar with practicable exercises.

4) Tobacco and stimulants: besides cigarettes and alcohol, other locally accessible stimulants like snuff (fine-ground tobacco) and kola (caffeine-containing) nuts are also used by some people in the region. Some patients perceive positive effects from using these substances, as illustrated in the following expressed views: cigarette is useful in relieving stress/tension; palm wine (local alcoholic beverage) and *Ogogoro* (distilled palm wine) make vision clearer; snuff stimulates work; and eating kola nuts is ideal norm for elders. A 65 year old man with controlled hypertension [ID23] responded – *our fore fathers used to say palm wine makes one to see very well, but it is glaring to me now that such thing is not good*. Another elderly (70 year old) man with uncontrolled hypertension [ID29] said – *in this environment, we believe kola nuts and snuff aid work, though I personally don’t use any of them*. A 60 year old woman with controlled hypertension [ID33] said – *I still eat Kola nuts although I have reduced the quantity I consume since I became hypertensive.* Such views make it difficult to quit using these substances. Another 64 year old man with controlled hypertension [ID25] highlighted the social consequences of giving up smoking and drinking thus – *the people and friends I used to drink and smoke with had to separate from me gradually after I stopped these habits, but this is fine with me as my health is more important.*

Table 5 shows perceived facilitators for implementing behavioral measures.

**Table 5:** **Factors facilitating compliance with behavioral measures: thematic matrix of categories, subcategories and concepts (n = 40)**

A) Salt reduction		
Category	Sub-category	Concepts
1) Health education	Multiple educational channels	Information from extra channels (radio, church, mosque) reinforces Doctor’s effort [n = 9]*
	Relevance and local content of education messages	Counseling on available suitable local substitutes for salt, maggi – Iru ‘Locust beans’ paste [n = 9]
		Counseling on ‘saltiness’ of meals not self-prepared – (food from canteens, social ‘parties) [n = 4]
2) Local practices	Compliance easy with substitutes	‘Iru’ cheap, easy to find [n = 9]
3) Social support factors	Family cooperation	Readiness of other family members to adjust to meals prepared with less salt [n = 26]
B) Weight control		
Category	Sub-category	Concepts
1) Local practices	Perception of body size; weight and beauty	Societal view that being too fat means ugliness, sexual unattractiveness [n = 5]
	Perception of body size; weight and body smartness	Perception that being too fat leads to physical unfitness (can’t lift body) [n = 3]
	Vegetable gardening and farming practice	Vegetables cheaply available and easily grown [n = 4]
	Possibility and practice of fishing (from local rivers)	Fish easily available and more consumed than meat [n = 7]
C) Exercising		
Category	Sub-category	Concepts
1) Social support factors	Awareness that exercise requires not much extra effort such as:	Possible to exercise using everyday activities [n = 11]
	using household chores to exercise	Sweeping, washing clothes, Pulling water from well [n = 10]
	farming to exercise	Hoeing, shoveling, clearing bush, harvesting, gardening [n = 4]
	transporting to exercise	Canoe paddling, bicycling, walking [n = 23]
	preparing food to exercise	Mortar grinding/pounding, wood axing [n = 11]
	exercising during religious worship practice/meetings	Clapping, dancing, singing, jumping, bending & rising [n = 5]
	using leisure to exercise	Drumming, cultural dancing [n = 11]
2) Patient related factors	Perceived influence of exercise on hypertension	Exercising makes body light and good for BP control [n = 27]
3) Health education	Reinforcement through education	Exposure to regular counseling on need to exercise [n = 19]
D) Quitting/not using tobacco, alcohol, stimulants		
Category	Sub-category	Concepts
Religion related factors	Faith based support (health counseling)	Abhorrence of ‘ungodly’ (unhealthy) social habits (smoking, alcohol use) by Islam and Christianity [n = 2]
Social support factors	Gender based support (societal view)	African society frowns at the habit of women smoking or using alcohol [n = 2]

*n refers to the number of respondents whose perceptions contribute to the corresponding concepts.

1. Salt: some patients mentioned that ‘health education’ had facilitated the reduction of their use of salt. Respondents highlighted the usefulness of getting health education from multiple ‘channels’ such as churches and mosques, besides the doctor, and of getting information about suitable local substitutes for salt and *maggi. Iru* (African locust bean paste) is a harmless locally available substitute for salt; it has similar taste as salt, is natural and normally contains no added sodium. A 49 year old female respondent with controlled hypertension [ID14] stated – *I got useful advices and was able to apply them though with little difficulty; I later (…) replaced salt and maggi with Iru which serves same purpose although I have also been using Iru together with salt before now.* The availability of substitutes for salt in local markets was similarly viewed as a facilitator. Finally ‘social support’ particularly the family’s willingness to eat low salt meals was identified as an important motivator for reducing salt. As one respondent, a 50 year old woman with controlled hypertension [ID11] puts it – *yes, I was able to make use of the advice; we now reduce the quantity of salt added to common ‘family pot’ food generally; thereafter those in the family that desire more salt in their food can add extra salt to their portion after dishing.*
2. Weight control: the perceived facilitators of weight control related mostly to local practices. Some people said the changing local and cultural perceptions on linkages between: ‘weight, wealth, beauty and health’ are an important condition to facilitate weight control. Being too fat is sometimes viewed as being tantamount to ugliness and physical unfitness or sluggishness. When asked what people’s comments on fatness are, a 65 year old man with controlled hypertension [ID23] replied – *people will say, look at him, ‘big for nothing’, yet he will not be able to enjoy breathing, worse still, his bigness really has nothing to do with wealth.* Another respondent [ID26] said – *beauty is a good thing that sometimes goes with bigness but some people are ugly looking when fat.* The ample availability of affordable healthy foods from local vegetable farmers and fishermen was equally seen as an important motivator for changing one’s diet and for weight control.
3. Exercise: some respondents saw their regular daily activities as good and socially acceptable ways for getting enough exercise without much extra effort. A 63 year old man with controlled hypertension [ID30] said – *exercise is generally good but the only ways I exercise is to walk to the farm, work on the farm and walk back home. I do walk regularly where I sometimes can otherwise use transportation.* Another woman [ID33] said – *exercising is good for hypertension; although I can’t farm, I exercise by washing clothes in the river and axing woods to make fire for cooking.* A 55 year old woman with uncontrolled hypertension [ID16] said – *I was told to do some exercise every morning like doing some domestic chores, walking (…); also, dancing and clapping in church are other kinds of exercise although we don’t normally clap in my church but we dance and sweat in the process.* Alluding further to the usefulness of household chores in exercising, an elderly male respondent with controlled hypertension [ID21] said – *in my community, women pound yam using mortar to prepare pounded yam, a popular local delicacy; this is also a form of exercise because they sweat a lot when they grind the mortar.* Another respondent, a 57 year old man with uncontrolled hypertension [ID26] said – *I get some fitness while paddling my canoe on the river in the mornings while fishing or transporting; this can also serve as exercise. I don’t have time for extra activities.*
4. Tobacco and stimulants: social support was perceived as the most important facilitator for cessation of smoking and reduction of the use of alcohol and other local stimulants. This category is sub-divided into supports people get from ‘faith-based’, and ‘gender-based’ social norms. These habits are seriously frowned upon and discouraged by the two major religions – Islam and Christianity. A 56 year old female respondent with controlled hypertension [ID37] stated – *I have been advised about the danger of all these things (smoking, alcohol, snuff, kola nut) but I don’t even do them before; my religion does not allow me to use them.* Similarly, there exists a prevailing gender-based perception or norm in most African societies that a woman should not smoke cigarette or drink alcohol, although this has become less stringent nowadays.

## Discussion

In this study we explored perspectives on treatment adherence among patients who received hypertension care in the context of a community based health insurance program in rural Nigeria. Results suggest that, having free access to previously unaffordable high quality care, from a healthcare center headed by a physician who was highly respected in the community, and to Western medication, were perceived as important facilitators for being adherent to medications, as was the perception that hypertension is a serious condition. Similarly, the performance of prayers, support from family members and other patients with hypertension and counseling from religious institutions were seen as factors reinforcing adequate medication use. Interestingly, compared to results from similar qualitative studies on hypertension in Western countries [22] the patients in this study regarded modern “white man’s” medications more positively and seemed to be less likely to alter the prescription according to their own insights. This finding seems credible in the light of a number of other Nigerian studies. In a qualitative study of explanatory models of hypertension among patients in a Lagos hospital, it was found that patients lacked “the desire to be active participants in their health care decisions” while they believed that “the doctor knew what was best for their care” [33]. Another study from urban Western Nigeria also found that patients were motivated to comply with treatment because they had access to Western prescription medications [36]. The Nigerian antihypertensive adherence trial demonstrated that medication adherence was very high among those participants who did not drop out of the study. The authors concluded that this is in line with the general experience that patients with “no or little prior contact with organized modern medical care will readily adhere to recommended therapy” [38].

At the same time, however, certain characteristics of the way care was organized could still hinder medication adherence, the most important being travel costs associated with clinic visits for those patients living far from the clinic, clinic operating hours, waiting times and the under dispensing of prescribed medications. In addition, patients’ idea that hypertension is a temporary condition for which medications may no longer be required once symptoms disappear, was an inhibiting factor for taking medications regularly, or a reason for omitting clinic visits. While the practice of faith healing or the use of herbal drugs might inhibit the use of prescription drugs, in their own case, participants said they would use these remedies only to supplement antihypertensive medications and not as a substitute. Interestingly, lack of social support from the patient’s immediate environment was rarely mentioned as an inhibitor for using medications.

These findings are plausible in the light of other studies that have also identified travel costs, long waiting times, clinic operating hours and limited knowledge by patients as factors limiting medication use [16,33].

While most respondents said they were aware that healthy behaviors could have a positive influence on their health and BP, adopting a healthy lifestyle seemed to be a greater challenge to them than taking their medications. Local practices and norms held by people in the community were identified as important inhibiting factors for behavioral changes including: use of salt for food preservation; negative cultural images associated with decreased body size and physical activity; and perceived (social) benefits of using palm wine, kola nuts, tobacco and snuff. Anthropologists have noted that a resistance to change (traditional) local food and eating practices and body images is common among people throughout the world, as these are often seen as an important component of an individual’s cultural identity [51]. Yet, our study offers also some insight into the factors that may facilitate healthier behaviors, and tackle the inhibitors. These include sufficient knowledge or understanding of how healthy behaviors may affect hypertension, and support from family or religion, but most importantly the awareness that healthier behaviors will not require profound changes of one’s usual daily life.

By using open interviews, the strength of this study is that its builds on direct experiences of the patients. We interviewed a sufficient number of patients to achieve thematic saturation, which is a criterion for sample size and internal validity in qualitative studies [43]. The iterative approach taken in this study [44] and specifically the decision to collect additional data on the basis of themes that were unclear from the preliminary analysis of data from the first set of interviews, added value to the results. We learned from these additional interviews that our informants attached significant importance to a traditional rural African way of life. And, when probed, they were readily able to describe how this way of life, not only provides inhibitors, but also opportunities for adhering to the behavioral recommendations they were given by their doctor. For example, the interviews provided much detail on what affordable food products are available in local markets to compose healthy meals (certain types of fruits, salt substitutes, vegetables and fish), and how people’s normal daily activities (e.g. farming, household chores, yam pounding, walking, drawing water from the well) offer ample opportunities for getting exercise. The additional interviews also provided further insight into reasons for medication adherence. It became clear that some patients felt particularly motivated or obliged to adhere to their medications because they perceived their health insurance and, consequently, their access to good treatment as blessings from God. But at the same time, religious practices (e.g., fasting) could sometimes pose practical barriers to medication adherence.

In line with quality criteria of qualitative research [50], we undertook several efforts to reduce individual biases in the analysis and to strengthen the trustworthiness and credibility of the findings: e.g., by using MAXQDA software to ensure consistency of analytic procedures, and by involving two or more members of the research team at crucial stages of the analysis.

The study also has limitations. The participants consisted of insured hypertensive rural dwelling low income Nigerians reachable through telephone, who had continued to visit the clinic for treatment for at least one year. Consequently, our findings do not capture ideas from other groups of patients. For example, within the context of a community based health insurance program, it would also be relevant to further investigate views on treatment adherence among patients who dropped out of treatment, despite the fact that they had access to good care. In terms of data collection and analysis, our possibility to verify what patients told us against other sources (triangulation) was limited: ethical considerations prevented us from interviewing significant others to crosscheck information from participants. Similarly, since the interviews were conducted in local languages and transcribed in English, it is not impossible that some deeper meanings participants’ intended to convey might be lost in the process of translation.

Poor adherence to treatment has been identified as one of the most important modifiable barriers to BP control in patients with hypertension [14,19,52]. Overlooking the accounts of the patients who participated in this study, it becomes clear that adherence can be affected by multiple spheres of influence including public policy (e.g., health insurance), institutional and organizational factors (e.g., health care system), environmental factors (e.g., availability of healthy foods), social and cultural norms and practices (e.g., food conservation and preparation practices), social networks (e.g., interactions with other patients, family members) and intrapersonal factors (e.g., patients’ knowledge and awareness level).

This provides support for social ecological approaches to health promotion [53]. These approaches assume that single interventions, such as the provision of free access to care are generally not sufficient to promote healthier behaviors and that a mixture of interventions aimed at different levels is needed to accomplish this.

**Figure 1:**
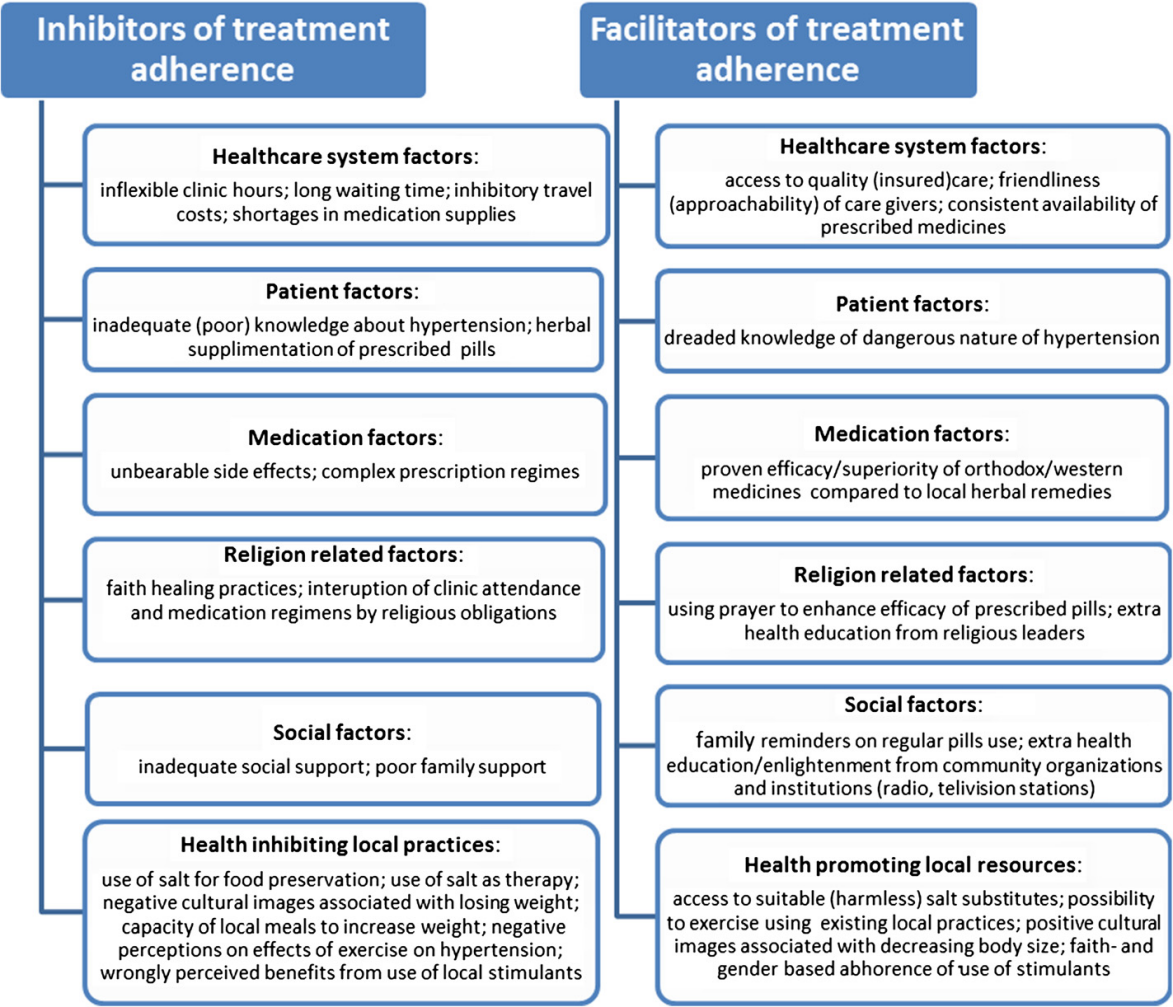
**Summary of factors related to hypertension treatment adherence.**

The framework of inhibitors and facilitators to adherence that emerged from this study (see Figure 1) suggests that, in the case of the community based health insurance program in Kwara state, a number of additional interventions may be needed to remove barriers that patients face in adhering to their treatment.

First, more attention could be paid to patient education. The findings suggest that patients with difficulty in managing their medications may benefit from further counseling or education about the nature of hypertension, why medications need to be taken regularly, what they may discuss with their doctor to make regular medication use easier (e.g., side effects, dosing, alternative medications, frequency of clinic visits), how they may deal with specific social, cultural or religious impediments encountered in taking their medications, and with the logistic challenges faced in getting refills on time.

In addition, patients who experience challenges in managing behavioral changes may benefit from further counseling or education about the positive influence of salt reduction, weight control, healthy diet and exercise on BP and health, and on how existing dietary and physical activity-related local practices may help them in implementing behavioral changes.

Community education may be one possible solution to avoid limited human resources and financial problems which patient education can pose to the already charged health care clinics. This type of education has been effectively used in the USA [54]. It can be delivered to groups of patients at the health care center or in other community settings the patients are familiar with, such as schools, churches or mosques. The education must be delivered by people who are known, trusted, culturally competent, and fluent in the language of the target community. These educators need not be health professionals, but they do need to be prepared for their role. Such preparation can be provided through careful training programs which can be offered by the health care center.

Secondly, it must be acknowledged that not all the patient-perceived barriers to adherence that were identified in this study can be tackled by education alone. Solutions will also have to be found at the level of the health care facility or the insurance program, for instance in order to address some of the logistic obstacles patients face with the monthly clinic visits.

## Conclusion

This qualitative study of patients’ perspectives on adherence to prescribed treatment reveals that hypertensive patients in an SSA setting who receive affordable care may still face challenges in adhering effectively to their prescribed hypertension treatment.

More than just ‘health insurance’ is required to enable adherence to treatment. Local communities in rural Africa may present specific barriers but also opportunities for adhering to medications and to healthier behaviors. With more insight into the specific inhibitors and facilitators perceived or experienced by patients, actionable community based educational interventions can be designed to strengthen adherence. However, some barriers cannot be tackled through patient education alone and should be addressed by interventions at the level of health care services and payment systems.

### Key messages

1. Despite ‘affordability of care’ – a feature of community based health insurance programs and insured care in general, hypertensive patients in SSA settings may still face other challenges in adhering effectively to prescribed treatment. More than ‘just’ health insurance is required to improve adherence and treatment outcomes.
2. Using information from exploration of patients’ perspectives on adherence to prescribed treatment this study suggests that local communities in low resource settings may offer specific barriers but also opportunities for adhering to medications and to healthier behaviors.
3. Adherence counseling and education for patients may benefit from addressing the identified specific inhibitors and facilitators perceived or experienced by patients.

## Additional files

Additional file 1:
**Interview guide used for QUICK-II study qualitative interviews with hypertensive patients.**
Additional file 2:
**Examples of coding steps and matrix for facilitators of treatment adherence.**

## Abbreviations

BP: Blood pressure
CVD: Cardiovascular disease
ID: Identity
MAXQDA: MAX qualitative data analysis
SSA: Sub Saharan Africa
