# Supplementary material for: Perceptions of inhibitors and facilitators for adhering to hypertension treatment among insured patients in rural Nigeria: a qualitative study
Source: BMC Health Serv Res. 2014 Dec 10;14:624. doi: 10.1186/s12913-014-0624-z (PMC4267751; doi:10.1186/s12913-014-0624-z)
Supplement: Additional file 1: — Interview guide used for QUICK-II study qualitative interviews with hypertensive patients. [file 12913_2014_624_MOESM1_ESM.docx]

**Interview guide used for QUICK-II qualitative interviews with hypertensive patients**

Preparation

-Greetings and familiarization

-Determine the respondent’s preferred language (Yoruba, English, Nupe) and address him/her in the chosen language.

-Explain the purpose of the interview and informed consent (briefly).

-Ask participant to provide informed and audio-record informed consent.

-Ask participant permission for audio-recording the interview.

-Take brief assessment of socio-demographic data (e.g., age, education, ethnicity, marital status, religion, employment status, income, health insurance).

Introduction

It is often difficult for care providers to give their patients advice on how to manage hypertension in their every-day life, if they are not familiar with the views and experiences of the patients themselves. For that reason, in this interview, I would like to ask you some questions so that we can learn more about patients’ views on hypertension and its treatment. There are no good or bad answers to the questions I will ask you. I would like to encourage you to tell me about your own personal views and experiences in your own words. You are welcome to ask me for clarifications if my questions are not clear. Everything you say will remain confidential. If we report on these interviews, we will not use your name or any other information by which you can be identified.

Questions

*Knowledge and personal views on hypertension*

1. When were you first diagnosed with hypertension? By whom and where?
2. What is hypertension, in your view; how is it called in your language?
3. What do you think can cause hypertension?
4. What do you think has caused your own hypertension?
5. What does it mean to have hypertension
   1. Do you notice anything, for example signs or symptoms or other things?
   2. How do you react in those cases?
   3. Has the hypertension affected your daily living, social life, religious and other activities? If so, can you tell me how?
6. How do you think your hypertension will progress further from now?
   1. Are there any dangers?
7. For how long do you think you will continue to seek treatment in the hospital for hypertension?
8. Are there any other ways (apart from the care you get in this hospital) in which you have sought treatment for your hypertension since diagnosed?

*Access to care*

1. How often do you consult your Doctor for hypertension care?
   1. Do you think this frequency is sufficient, or too little or too much?
2. Can you tell me something about the time it takes to access treatment?
   1. How much time do you spend in traveling to clinic?
   2. What is the total time you spend in clinic accessing care?
3. Can you tell me something about the costs involved in your treatment
   1. Travel costs you incur in attending?
   2. Any other costs?

*Health insurance issues*

1. When and why did you enroll in the health insurance program?
2. How do you view the Hygeia Community Health Care insurance program generally, and particularly with regards to your hypertension care?

*Information and sources*

1. Apart from what you already knew, what information/advice did you get from your doctor(s) about hypertension, its effects, and dangers and how to live positively with it?
2. Did you seek or get such information also from other source(s)?
   1. If any, from which sources? And what type of information?

*Views on (prescribed) medications*

1. What kinds of medication are usually prescribed to you by your doctors? How often do you have to take them?
2. How carefully / regularly do you want to take your prescribed medicines?
3. How is your pill taking actually going now?
   1. How often do you forget to take your medicines?
   2. Are there any difficulties in taking your medicines on time?
      1. probe: availability, quantity, side effects, religious/faith related practices – occasionally abstaining from food/all other ingestions to *fast* for several hours/days; effect on body and blood pressure, when traveling
   3. If so, how have you been coping with such difficulties?
4. Apart from prescribed medications, are there any other medications/remedies you also take for hypertension?
   1. If so, why and how often do you take them?
   2. If so, how do you get these remedies?
   3. Do you combine these medications with your prescribed pills?
   4. Why, why not?
5. How long do you think you will have to continue to take your hypertension medicines (temporarily or forever)?
6. In general what benefits or disadvantages do you see in the regular application of your prescribed or other medicines?

*Views of behavioral adaptations*

1. Did you get any recommendations from your doctor (or others) about what you can do yourself in order to live positively with hypertension (diet, exercise)
   1. Please explain?
2. In your view, how can any of the following behaviors affect hypertension: physical exercise, reduction of salt use, a healthy diet, reduction of overweight/body size?

Please discuss any of these behaviors

- 1. What might be the influence of the behavior on hypertension?
  2. Do you engage in any of these behaviors? If so, which?
  3. What makes the behavior(s) possible or difficult?
  4. How do people in the community regard the behavior?
  5. What are tips/experiences of actions that would permit a healthier behavior, if any?

1. In your view, how can any of the following customs/behaviors that people may engage in affect hypertension: smoking (tobacco), use of alcohol/palm wine/Ogogoro (local gin), snuff/Taba (fine ground tobacco) inhalation, and consumption of Kola (caffeine-containing) nuts?

Please discuss any of these behaviors

- 1. What might be the influence of the behavior on hypertension?
  2. Do you engage in any of these behaviors? If so, which?
  3. What makes the behavior(s) possible or difficult?
  4. What can prevent the behavior?
  5. How do people in the community regard the behavior?
  6. What are tips that might help refrain people from the behavior, if any?

1. Are there any other issues in relation to behavior and hypertension you would like to mention/discuss?

*Doctor –Patient Relationship*

1. When last did you see your Doctor in the last 12 months for hypertension care? How regularly do you attend your follow-up visit appointments?
2. How do you find communication with your Doctor in the treatment of your hypertension?
3. How comfortable are you with your current doctor? How much do your doctors currently satisfy your expectations in taking care of your hypertension? How do you view the attitude of other care givers e.g. nurses, pharmacists towards you in caring for your hypertension?
4. How much room or opportunity are you given during consultations to express your feelings about your treatment and high blood pressure?

*General view on hypertension and cardiovascular diseases prevention care(if not discussed before)*

1. In your opinion, what factors can facilitate good hypertension care and prevention of its complications in this community?
2. In your opinion, what issues militate against your access to good hypertension care and prevention of its complications in this community?

**Note**: This *guide* contains the broad topic areas that were addressed during the interviews. As is common in qualitative interviews, questions were sometimes modified to address the specific concerns and answers of participants. In addition, after a number of interviews were completed, more specific information became available, particularly about health behaviors (question 22-25), so that more specific questions could be asked during the follow-up interviews.
