# Supplementary material for: Perceptions of inhibitors and facilitators for adhering to hypertension treatment among insured patients in rural Nigeria: a qualitative study
Source: BMC Health Serv Res. 2014 Dec 10;14:624. doi: 10.1186/s12913-014-0624-z (PMC4267751; doi:10.1186/s12913-014-0624-z)
Supplement: Additional file 2: — Examples of coding steps and matrix for facilitators of treatment adherence. [file 12913_2014_624_MOESM2_ESM.docx]

**Examples of coding steps and matrix for facilitators of treatment adherence**

| **Participants’ statements** | **Assigned code** | **Concept** | **Sub-category** | **Main category** |
| --- | --- | --- | --- | --- |
| 1. Prescribed medications | | | | |
| *- I am into herbs and I have used them severally in the past but they did not work like the white man’s medicine* | Adherence enabling strategies ^CODE 2^  (medications) | White man’s pills work better than traditional medicines (herbs) | Perceived efficacy of orthodox medicines | Medication related factors |
| *- If the pills can be formulated such that fewer pills will perfectly do the work of the 8 pills that I currently take daily; then fewer pills are preferred as they are easier for me to manage* | Adherence enabling strategies ^CODE 2^  (medications) | The fewer the pills, the easier to adhere to prescription | Simplicity of prescription regimes |  |
| 1. Behavioral recommendations | | | | |
| *- They do advise us on hypertension in my church, like advice that we should reduce salt, magi and starchy food like the Doctor use to tell us. I put all the advice into use* | Possibilities from existing local practice ^CODE 2^ (healthy behavior) | Motivation from health counselling at religious meetings / services | Faith based support |  |
| *- Although I have not yet been advised about these things (smoking, alcohol, snuff) but I can’t even think of doing them at all; even my husband who is a man does not use these things talk less of me - a woman* | Possibilities from existing local practice ^CODE 2^ (healthy behavior) | African society frowns at the habit of women smoking or using alcohol | Gender based support | Social support factors |
| *- Based on advice, I later replaced salt and Magi with a local seasoning - Iru (locust beans paste) which serves same purpose as salt and contained no salt* | Possibilities from existing local practice ^CODE 1^ (diet) | Local availability of suitable substitutes to harmful diet | Environmental support |  |
